# The Muzzling Order and Its Effects

**Published:** 1897-04-24

**Authors:** 


Vol. XXII.—No. 552.] [April 24, 1897.

### The Muzzling Order and its Effects.

Few things can be more curious, to the student of sociology, than the manner in which the order for muzzling dogs, recently issued by the Board of Agriculture, has been received by a small but noisy section of the public. There is a well-known story of a great lady to whom the intelligence was brought that her favourite lap-dog had bitten one of the footmen, and who exclaimed with the most lively sympathy, "Oh! poor thing! I hope he won't be sick!" This is precisely the mental attitude of the self-styled "dog-lovers" who have lately inundated the Press with silly letters about the sufferings of their pets, some of whom—if the owner's tales be true—have positively had their noses scratched, or even their foreheads galled by the wires of muzzles which the aforesaid owners had not taken the trouble to obtain of proper size and shape. No one who frequents any of the open spaces or recreation grounds which abound near London can fail to see that, in a very short time, the average dog is at least as little inconvenienced by his muzzle as the dog's owner is by his hat, and is far less inconvenienced than a horse must be by his headstall and bit. Such is the magnitude of the grievance on account of which some of the letter writers are prepared to abandon what they are pleased to call their political principles, and are longing for an opportunity of voting for Parliamentary candidates who, if they will but vote against the muzzle, may do as they like with the concert of Europe, and may light, without thought as to how or when it would be extinguished, the torch of war. It is charitable to hope that such people have as little knowledge of the horrors of war as they have of the horrors of hydrophobia.

According to the returns of the Registrar General, in the 25 years ending with 1872, 373 persons, 299 males and 74 females, died in England and Wales from hydrophobia, or almost exactly 16 persons a year. All these persons had been bitten by rabid dogs; and Dr. Gowers, in an account of the disease based upon so considerable an experience that he has himself made *post mortem* examinations of the bodies of nine victims, asserts that, of those bitten by certainly rabid dogs, 47 per cent. have suffered; of those uncauterised, 83 per cent.; and of those promptly cauterised, only 33 per cent. On the other hand, of persons bitten by dogs merely suspected of rabies, only 8 per cent. have suffered. The immunity of some individuals, he adds, has perhaps been due to the teeth of the dog having been cleaned by passing through clothes, or by immediately preceding bites, but the poison probably varies in virulence, and it is possible that there are differences in susceptibility in the human subject, such as apparently exist in dogs. To take more recent figures, and to show that the risks of being bitten have not undergone any diminution since 1872, we may say that, in the nine years terminating with 1895, 17,337 persons were inoculated at the Pasteur Institute in Paris. All of these had been bitten, but not all by dogs, as there were many cases of wolf bite from Russia. In 1895, 1,520 persons were inoculated at the institute, of whom 122 had been bitten by animals experimentally proved to be rabid, 949 by animals declared by veterinary certificate to be rabid, and 449 by animals supposed to be rabid. Out of the whole number there were only two deaths from hydrophobia; but although Pasteur's discovery has largely robbed the bite of a rabid animal of its dangers, we are not thereby released from the duty of endeavouring to free the canine species, and mankind, from what has hitherto been a scourge to both. The Pasteur Institute is not very accessible to English people, especially to the poor and their children in rural districts; and it would be eminently desirable that all occasion for having recourse to it should be set aside. There is an absolute consensus of scientific opinion to the effect that rabies, and consequently hydrophobia, could be completely stamped out in England if a general order for muzzling were enforced for a period of from six to twelve months, and if all ownerless dogs were destroyed; while the experience of Australia, where neither disease has ever occurred, shows that neither has any tendency to arise spontaneously. When Mr. Long addresses his constituents, prior to the reassembling of Parliament, he will be likely to explain his intentions in the matter, and to make public the advice by which he is being guided. It is said that districts not at present included in the order will be scheduled before long, but that, in order to give time to the manufacturers of muzzles, it was expedient to begin with selected localities, and that there may even be some in which rabies has never shown any tendency to prevail, and which may safely be permitted to remain exempt. We doubt whether any such exemptions would be either justifiable or expedient, and they could hardly fail to furnish occasions for clamour to the shriekers, a consideration not to be wholly neglected by a prudent Government. What may be called "Anti-cism," of whatever kind, does, indeed, usually originate with fools, but in no long time people of more astuteness are apt to see money in it, and to organise some kind of profitable agitation for its support. We have noticed a few feelers in this direction with regard to the muzzle, but are not without hope that the common sense of the public will disregard them.

